# THE ROLE OF COMMUNITY COLLEGES IN PARTNERSHIPS WITH UNIVERSITIES IN PRODUCING QUALIFIED GERONTOLOGY PROFESSIONALS

**DOI:** 10.1093/geroni/igz038.903

**Published:** 2019-11-08

**Authors:** Karen Kopera-Frye

**Affiliations:** New Mexico State University, Las Cruces, New Mexico, United States

## Abstract

Today is especially critical for community colleges and universities to work together to allow for pathways in educating professionals in the field of gerontology. At New Mexico State University, Public Health Sciences Department, we have partnered with Dona Ana Community College and Central New Mexico College through articulation agreements to allow transfer of credits and an accelerated pathway to a Gerontology Minor for workforce development. This paper will discuss the steps we took in bridging these educational opportunities as an example for other institutions to consider. By incorporating joint Advisory Boards, faculty and administrators entering into articulation agreements, and coordinated course offerings, we were able to systematize the curriculum and allow for transfer of up to 12 credits from the community colleges into our Public Health Bachelors degree program, with attainment of the Gerontology minor along the way. Lessons learned during this process will be shared.

